# The Leucocyte in Disease

**Published:** 1896-03-14

**Authors:** H. S. Wansbrough Jones


### Medical Progress and Hospital Clinics.

[*The Editor will be glad to receive offers of co-operation and contributions from members of the profession. All letters should be addressed to* The Editor, at the Office, 428, Strand, London, W.C.]

### THE LEUCOCYTE IN DISEASE.

By H. S. Wansbrough Jones, M.B., C.M.Edin.; B.Sc.Lond.

The history of blood research since the time of Wooldridge is the history of the exaltation of the leucocyte; ascertained facts have raised it to such a position of importance that one is almost tempted to regard the old Levitical saying, "the life of the flesh is in the blood," as capable of literal and scientific interpretation. Before presenting what appear to me to be the most interesting aspects of the action of these bodies in diseases, it will be necessary to give a short *résumé* of our present knowledge of their morphology, physiology, and chemistry.

Ever since the publication of Ehrlich's paper on "The Staining Reaction of Leucocytes" many varieties have been recognised, differing from each other not only in their affinities for special dyes, but also in their functions, and probably also in their origin. Many classifications have been attempted, but the following, given by Kanthack, agrees in the main with that of Ehrlich, but is simpler and less arbitrary. It differs, however, from Ehrlich's in the use of the term "finely granular eosinophile cells," instead of "neutrophile." Kanthack points out that Ehrlich's so-called neutral dyes, which are a mixture of acid and basic dyes in certain proportions, act in reality as acid dyes, and that the granules of Ehrlich's neutrophile cells stain readily with eosin if the blood-films are dried quickly and stained immediately. Sherrington and some of the other English investigators agree with Kanthack. I myself have never had any difficulty in demonstrating the eosinophile affinity of the fine granules in these cells, which form about 75 per cent. of the total number of leucocytes in a normal specimen. In blood-film, dried quickly and stained first with eosin (acid dye) and then with methylene blue (basic dye) the following varieties of leucocyte may be found: (1) Lymphocytes (15—25 per cent.), small cells; large blue nucleus; protoplasm clear. (2) Large uninuclear cells (6 per cent.); larger cells, oval or indented light blue nucleus; relatively large clear zone of protoplasm. (3) Coarsely granular eosinophile cells (1—5 per cent.); single round or horseshoe-shaped nucleus, stained blue; protoplasm filled with large granules stained with eosin. (4) Finely granular eosinophile cells (70—75 per cent.) (Ehrlich's neutrophile); about same size as 3; nucleus lobed or multipartite (hence sometimes called multinuclear cells); protoplasm filled with fine granules stained with eosin. (5) Basophile cells or mastzellen; very rare in human blood; found in connective tissue; nucleus blue; protoplasm granular; granules stained purplish blue. All have the power of amœboid movement and migration, and all except the coarsely granular eosinophile and the basophile cells are phagocytic, *i.e.*, possess the power of taking up foreign particles and digesting them. It is of course known also that they can take up indigestible foreign particles, like coal dust, from the lung, carry and deposit them in the lymphatic glands. As regards their origin there is still much uncertainty, and it is not yet known whether they should all be regarded as distinct morphologically, or only as different stages in the life history of the same individual.

Certainly there is an intimate relation between the lymphocytes and the lymphglands; the large uninuclear cells and the spleen and marrow; and between the basophile and coarsely granular esinophile and the connective tissues generally. It is probable also that when once they have appeared in the blood they do not change into other forms, for no definite transition stages have ever been seen (Kanthack). At the same time it is certain that they can multiply within the blood, for not only are karyokinetic and mitotic changes in the nucleus frequently seen, but Römer, who found that injections of Buchner's protein caused an increase in the number of leucocytes in the blood of rabbits, discovered also by cutting off the ears after injection elsewhere, and keeping them in a warm moist chamber, that this leucocytosis goes on even after the ear is separated from the body

The proportion of leucocytes to red corpuscles in the blood is normally about 1 : 500. This is increased in the first two hours after a meal to 1 : 150 or 1 : 100, the lymphocytes and finely granular eosinophile cells being increased, the coarsely granular eosinophile and large uninuclear cells decreased in number (Okintschitz).

There is leucocytosis in infancy (especially in eosinophile cells), and also during pregnancy.

During starvation there is diminution in number, with relative increase of the coarsely granular, and decrease of finely granular and lymphocytes. With a diminution in number there is probably an actual disintegration and solution of the leucocytes in the blood plasma, but this will be more fully discussed in the sequel.

Leucocytes contain, in addition to proteid material, lecthin and other fats, glycogen, and inorganic salts, especially potassium salts and phosphates. It is not, however, yet known whether these are essential constituents of the leucocyte, or have relation only to its function as food-carriers. The proteid constituents consist of paraglobulin and a cell-fibrinogen or nucleo-albumen, *i.e.*, a substance which differs from albumens in that it deposits a precipitate of nuclein on peptic digestion. The importance of this will be further emphasised in the second of these papers.

The function of the leucocyte in health is still obscure. It has been shown by Pohl that many tonics and stimulants cause a marked increase of leucocytes. Hofmeister long ago stated that the only way he could conceive of the passage of peptone into the blood or chyle was that it was taken up by the leucocytes. In confirmation of this view may be urged Wooldridge's observation that the weight of leucocytes in a measured volume of blood is increased after peptone injection, and also the well-known fact that the breaking down of leucocytes, whether in the formation of pus or other ways, is always accompanied by the appearance of peptone or albumose in the urine. It may be safely said, then, that the leucocyte is an organ of metabolism, and at the same time a food-carrier.

In considering the action of the leucocyte in disease attention will be specially directed to those phenomena which explain something of the process, symptoms, or results of disease, rather than to mere statistical observations as to number and variety.

It is proposed then to discuss (1) the phenomena connected with the presence of poisons in the blood; (2) some of the facts recently established with regard to the reaction of leucocytes to local irritants; (3) the fate of the leucocyte.

1. Incidentally reference was made above to Römer's experiments on the injection of Büchner's protein. There is an enormous quantity of literature on this subject, but all writers are apparently agreed that the effect of injecting physiological or bacterial poisons into the blood is a diminution in the number of leucocytes, followed, if the animal recover, by a leucocytosis. The following are some of the substances for which this fact has been established: Quinine (Binz), atropine and nuclein (Horbaczewski), peptone and tissue fibrinogen (Wright), leucocytes (Groth), curara, tuberculine, and other bacterial products, snake venom. It has been shown that the increase is chiefly in the finely granular eosinophile cells, and Kanthach asserts that their granulations stain more deeply. Where the injections are accompanied by fever the leucocytosis is simultaneous with the fall in temperature which follows the initial rise.

As the result of these observations the subject has been investigated in regard to the specific fevers. Leucocytosis has been observed in scarlet fever and diphtheria, in suppuration, pneumonia, pleurisy, and erysipelas. It does not occur in typhoid unless there is suppuration (V. Jaksch). Special interest attaches to the observations made in pneumonia, for both Von Jaksch and Kanthack state that they have been able to prophecy the fatal termination of a pneumonia from the fact that leucocytosis was absent. In all these conditions the increase is chiefly in the finely granular eosinophile cells. The leucocytosis of pneumonia is most marked at the pre-critical period; indeed, it is found that with the fall of temperature the number is markedly diminished, though it subsequently rises again.

Now it appears to me that the chief interest of these facts lies in the possible explanation they afford of some phenomena of inflammation and the meaning of immunity. We must suppose that the result of the taking in of the virus of a disease is to produce a profound and immediate effect on the activity of the leucocytes. No initial diminution in the number has yet been observed clinically; it may or may not occur. At any rate, if the vitality, shall we say, be sufficient, the leucocytes proliferate, and, whether by their action on the actual germs or the bacterial products, succeed in counteracting their baneful effects. Then comes another stage. The leucocytes, possibly themselves exhausted by their struggle, break down, fibrinogen is liberated, which further splits up into an albuminous moiety and nuclein moiety, and appears in the urine as albumose and uric acid.

But I think we may go a step further in legitimate speculation on this subject. Let us look at the facts ascertained in regard to pneumonia, a pre-critical leucocytosis, a diminution at the crisis. Wright has observed that consolidated pneumonic lungs yield large quantities of tissue fibrinogen; and both he and some of Schmidt's pupils have shown that the injection of this substance is followed by a marked fall in the number of leucocytes. May it not be the case that with the commencing resolution of the lung this fibrogen is liberated, and acts on the leucocytes in this way, so that the first effect of the absorption of the exudation is to destroy the leucocytes, whose help is no longer needed; and it is not improbable that the weakness attending a crisis is due to the same destruction of leucocytes.

As regards immunity, Everard, Demoor, and Massart have shown that the inoculation of guinea pigs with pathogenic organisms is followed by a diminution in the number of leucocytes, and a subsequent leucocytosis; if, however, the animal be previously immunised, the leucocytosis develops at once. This might be interpreted to mean that in the process of immunisation the destruction of leucocytes frees some substance which, acting on the leucocytes subsequently developed in the animal, alters either their chemical constitution or their physical state, so that the injection of pathogenic organisms can no longer destroy, but only stimulate to increased activity. That this is no mere gratuitous hypothesis is shown by the fact that both Wooldridge and Wright have demonstrated that animals may be rendered immune from anthrax by the injection of tissue-fibrogen prepared in a special manner, but without the least contamination with anthrax, thereby establishing that immunity is due to chemical relations of the blood. My contention is, then, that where a destruction of leucocytes occurs as the result of the inoculation of the organisms of a disease, the liberation of fibrinogens so effected renders the blood uncongenial to organisms of the same nature, and protects the leucocytes of the future from a similar death.

(*To be continued.*)

